# Physician Perception of Fontan Failure, “Acceptable” Hemodynamics, Assessment, and Indications for Intervention—Results of a Multinational Survey

**DOI:** 10.1016/j.cjcpc.2025.02.004

**Published:** 2025-03-14

**Authors:** Ashish H. Shah, Evan J. Wiens, Jonathan Windram, Shakeel A. Qureshi, Petra Jenkins, Isma Rafiq, Erwin Oechslin, Richard A. Krasuski

**Affiliations:** a Department of Cardiology, St. Boniface Hospital, University of Manitoba, Winnipeg, Manitoba, Canada; b Department of Cardiology, University Hospital of Alberta, Edmonton, Alberta, Canada; c Department of Cardiology, Evelina London Children's Hospital, Guy’s and St Thomas’ NHS Foundation Trust, London, United Kingdom; d Department of Cardiology, Liverpool Heart and Chest Hospital, Liverpool, United Kingdom; e Department of Cardiology, Royal Brompton Hospital, London, United Kingdom; f Toronto Adult Congenital Heart Disease Program, University Hospital Network, and University of Toronto, Toronto, Ontario, Canada; g Division of Cardiology, Duke University Health System, Durham, North Carolina, USA

## Abstract

**Background:**

Patients with complex congenital heart disease unfit for biventricular repair are often palliated with Fontan circulation (FC), which obviates the need for a subpulmonary ventricle. This approach has led to high survival rates, with over 80% of patients expected to live beyond 30 years. Despite increasing patient numbers and existing guidelines, there are no standardized hemodynamic parameters defining Fontan failure, resulting in considerable variability in management practices. This pilot project aimed to assess real-world practices in FC patient management, particularly in surveillance, defining Fontan circulatory failure, and determining treatment thresholds.

**Methods:**

A cross-sectional survey with 10 multiple-choice questions and optional free-form responses was distributed globally to congenital heart disease cardiologists through 2 academic centers. Conducted from January to December 2021, the survey targeted clinicians managing adult FC patients.

**Results:**

Of 310 invited cardiologists, 170 (55%) participated, including 27% from the United States, 22% from Canada, and 20% from the United Kingdom. Respondents included pediatric (37%), adult congenital (47%), and dual trained adult/pediatric cardiologists (14%), mostly in academic settings (94%). Variability existed in defining FC failure, with markers such as protein-losing enteropathy (74%), fatigue/dyspnea (62%), and elevated Fontan pressure (58%) commonly cited. Responses differed on defining elevated Fontan pressure, with 53% selecting >15 mm Hg, 33% >18 mm Hg, and 14% >20 mm Hg. Ninety-one percent prescribed pulmonary vasodilators, though indications and thresholds varied.

**Conclusion:**

Substantial heterogeneity in FC patient management currently exists, underscoring the need for standardized hemodynamic parameters.

## Graphical abstract

**Figure:**
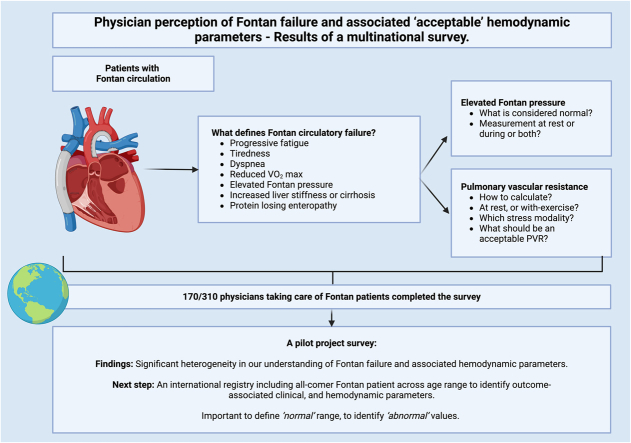

## Résumé

**Contexte:**

Les patients atteints d’une cardiopathie congénitale complexe chez qui la réparation biventriculaire est exclue en raison de leur condition physique subissent généralement une intervention chirurgicale palliative aboutissant à la création d’une circulation de Fontan (CF), donc sans ventricule sous-pulmonaire. Cette stratégie s’est traduite par des taux de survie élevés; plus de 80 % des patients peuvent espérer vivre plus de 30 ans. Malgré l’augmentation du nombre de patients et les lignes directrices existantes, il n’existe aucun paramètre hémodynamique normalisé pour définir l’échec de la CF, de sorte que les pratiques de prise en charge sont extrêmement variables. Ce projet pilote visait à évaluer en situation réelle les pratiques de prise en charge des patients ayant une CF, en particulier la surveillance, la définition de l’échec et la détermination des seuils de traitement.

**Méthodologie:**

Nous avons réalisé une enquête transversale comportant 10 questions à choix multiples et des questions ouvertes facultatives auprès de cardiologues spécialisés dans les cardiopathies congénitales un peu partout dans le monde, par l’entremise de deux centres universitaires. Cette enquête a été menée de janvier à décembre 2021 et ciblait les cliniciens prenant en charge des patients adultes avec une CF.

**Résultats:**

Au total, 55 % (170) des 310 cardiologues invités ont participé, dont 27 % aux États-Unis, 22 % au Canada et 20 % au Royaume-Uni. Les répondants comprenaient des cardiologues pédiatriques (37 %), des cardiologues spécialisés dans les cardiopathies congénitales chez l’adulte (47 %) et des cardiologues spécialisés à la fois dans le traitement des adultes et des enfants (14 %), dont la plupart exercent dans des établissements universitaires (94 %). La manière de définir l’échec de la CF varie : les marqueurs fréquemment mentionnés sont notamment l’entéropathie avec perte de protéines (74 %), la fatigue/dyspnée (62 %) et l’élévation de la pression dans la CF (58 %). Les réponses différaient en ce qui concerne la définition d’une pression élevée dans la CF : > 15 mmHg dans 53 % des réponses, > 18 mmHg dans 33 % des réponses et > 20 mmHg dans 14 % des réponses. Quatre-vingt-onze pour cent des répondants prescrivent des vasodilatateurs pulmonaires, bien que les indications et les seuils varient.

**Conclusions:**

La prise en charge des patients ayant une CF est très hétérogène actuellement, ce qui montre la nécessité de disposer de paramètres hémodynamiques normalisés.

Patients born with complex congenital heart disease (CHD) not amenable to biventricular repair are palliated with the establishment of a Fontan circulation (FC). FC incorporates the available ventricle(s) into the subaortic position and passive filling of the pulmonary circulation by directly connecting the superior and inferior vena cavae to the pulmonary vasculature. This unique circulation leaves the patients without a subpulmonary ventricle. Although technically considered a palliative procedure, nearly 90% of patients survive childhood, and >80% of patients are expected to live for at least 30 years.^1^ Between 2001 and 2014 in the United States alone, 15,934 patients underwent Fontan procedures.^2^ It is estimated that worldwide there are nearly 70,000 patients alive with FC, with the number expected to double over the next 20 years.^1^ Despite the increasing prevalence of patients living with FC and guidelines, which recommend regular invasive hemodynamic evaluation throughout their lifetime,^3^, ^4^, ^5^ there are no well-established normal hemodynamic values or an objective case definition of Fontan failure.^6^ This has resulted in considerable institutional variation in the surveillance and management of FC patients. A recently published consensus statement has described standardized definitions of Fontan-associated comorbidities.^7^

Current real-world practices of cardiologists taking care of Fontan patients are not well known, including methods used for routine surveillance, how they define FC failure clinically and hemodynamically, and their thresholds and methods for its treatment. We therefore conducted a survey of cardiologists managing adults with FC to better define practice patterns and identify areas of consensus, heterogeneity, and need for future study.

## Methods

This pilot project involved a brief cross-sectional survey to assess physicians’ opinion of failing Fontan, related hemodynamic parameters, and management strategies. A simple survey consisting of 10 questions was developed (Table 1). All questions had multiple-choice responses, but respondents also had the opportunity to add free-form responses. The survey was distributed through the Toronto International Adult Congenital Heart Disease Academic Rounds and the Western Canada Adult Congenital Surgical Conference, Edmonton, email distribution lists to cardiologists specializing in CHD management. The survey was distributed twice, with a 6-week interval between distributions. The second email served as a reminder, explicitly asking only those who had not previously completed the survey to participate. All participants were experienced cardiologists actively participating in the care of patients with FC. The survey was distributed in January 2021 and remained open until December 2021. Participation was voluntary, and respondents were not asked to provide features that could be used to determine their identities. Responses we compiled and reported descriptively. No remuneration was provided for the completion of the survey. The Duke Institutional Review Board provided exemption from the institutional review board review to the study (Pro00117267).

**Table 1:** Questions comprising the survey distributed to cardiologists managing patients with Fontan circulation

Question 1	How do you define Fontan circulatory failure?
Question 2	If you have chosen elevated Fontan pressure as your answer, what do you consider to be elevated mean Fontan pressure?
Question 3	Do you use pulmonary vasodilator therapy in patients with Fontan circulation?
Question 4	What are the indications for the use of pulmonary vasodilator therapy?
Question 5	To calculate transpulmonary gradient, do you subtract pulmonary capillary wedge pressure or left ventricular end-diastolic pressure from mean pulmonary artery pressure to calculate transpulmonary gradient?
Question 6	Do you assess patients for exercise-induced hemodynamic change?
Question 7	If you answered yes to the above question, what method do you use?
Question 8	What is your area of practice?
Question 9	What is your clinic setting?
Question 10	What is your geographical location?

## Results

### Participants

A total of 310 cardiologists were invited to participate, 170 (55%) of whom responded. Forty-six (27%) respondents were from the United States, 37 (22%) from Canada, 34 (20%) from the United Kingdom, 21 (12%) from Europe, 16 (9.4%) from South America, and 15 (9%) from Asia (Supplemental Fig. S1). Sixty-two (37%) identified as “pediatric cardiologists” and 80 (47%) as “adult congenital cardiologists.” Six (2%) respondents identified as “noncongenital cardiologists” and 23 (14%) identified as cardiologists who managed both pediatric and adult congenital patients (Supplemental Fig. S2). Their location of practice was almost exclusively an academic or university setting (159, 94%), with 10 (6%) practicing in a private clinic (Supplemental Fig. S3).

### Definitions of Fontan circulatory failure

Respondents were allowed to select multiple responses to “How do you define Fontan circulatory failure?” The most commonly selected responses were “protein-losing enteropathy” (125, 74%), “worsening symptoms of fatigue, tiredness, and dyspnea” (105, 62%), and “elevated mean Fontan pressure obtained during catheterization” (100, 59%). Less than half of respondents (81, 48%) considered “evidence of increasing stiffness or liver cirrhosis” as a principal manifestation of Fontan circulatory failure, and 73 (43%) selected “reduced exercise capacity documented by reduced VO_2max_ on a cardiopulmonary exercise test” (Supplemental Fig. S4).

### Definition of elevated Fontan pressure

One hundred fifty-five (91%) of those surveyed provided a threshold above which they considered Fontan pressure to be abnormal; 83 (54%) identified this as >15 mm Hg, 51 (33%) as >18 mm Hg, and 21 (14%) >20 mm Hg (Supplemental Fig. S5). For the calculation of transpulmonary gradient, 80 (47%) subtracted the pulmonary capillary wedge pressure from the mean pulmonary artery pressure, 22 (13%) subtracted the left ventricular end-diastolic pressure, and 67 (40%) used either or both (Supplemental Fig. S6).

“Exercise-induced hemodynamic assessment” was used by 77 of 168 (46%) participants using various modalities including stress echocardiograms (40% exercise and 4% dobutamine), stress magnetic resonance (MR) imaging (11% dobutamine and 7% exercise), invasive hemodynamics using an exercise bike (20%), and cardiopulmonary exercise testing (0%) (Supplemental Figs. S7 and S8).

### Indications for pulmonary vasodilator therapy

Most respondents (155 of 170, 91%) used selective pulmonary vasodilator therapy in FC patients, with varying indications for this provided. One hundred twenty of 168 (71%) participants indicated that elevated mean Fontan pressure would be an indication, though disagreement existed about the threshold pressure to start therapy: 59 (49%) suggested >15 mm Hg, 37 (31%) >18 mm Hg, and 24 (20%) >20 mm Hg. Similarly, 118 (70%) indicated elevated pulmonary vascular resistance as an indication to start therapy, with disagreement on thresholds: 41 (35%) >2 Wood units, 59 (50%) >4 Wood units, and 18 (15%) >6 Wood units. Seventy-five (48%) of respondents started pulmonary vasodilators for protein-losing enteropathy; 64 (41%) for symptoms of dyspnea, tiredness, or worsening exercise capacity; 57 (37%) for reduced exercise capacity on cardiopulmonary exercise test; and 43 (28%) for objective evidence of hypoxia. Of the 23 (15%) who selected “other,” freeform answers suggested treatment based on a combination of symptoms, end-organ damage, and overall clinic status (Supplemental Figs. S9 and S10).

## Discussion

This survey of subspecialty cardiologists managing FC patients identified significant heterogeneity in clinical practice, including what defines Fontan circulatory failure, what should be considered an elevated Fontan pressure, whether hemodynamic assessment during exercise is necessary, and when to use pulmonary vasodilators. This is emblematic of the uncertainty in managing complex CHD, particularly the long-term complications of FC patients, and is related to a lack of high-quality evidence with respect to patient management.^1^

To develop and study potential therapies, identifying the target population is critical. Defining “normal” hemodynamic parameters, or the threshold values beyond which clinical risk increases, is a critical first step. A few cohort studies have attempted to identify hemodynamic parameters that predict outcomes in FC. One of the largest (548 patients, including 387 early group [0.5-5 years after Fontan completion] and 161 late group [>15 years after Fontan]) found that Fontan pressure >14 mm Hg and lower systemic oxygen saturation (hazard ratio of 1.14 per 1% reduction in saturation) predicted higher mortality. In the early group, additional mortality predictors included increased indexed ventricular end-diastolic volume and low ejection fraction, whereas in the late group, high cardiac output (>3 L/min/m^2^), especially when associated with Fontan pressure >14 mm Hg, resulted in higher mortality.^8^ Others have identified a high-risk phenotype with normal cardiac output and high Fontan pressure.^9^ However, the values used to define the “normal” range of cardiac output and Fontan pressure have been inconsistent.^8^^,^^9^ A recently published consensus statement identified an indexed pulmonary vascular resistance (PVR) (Wood units m^2^ body surface area) >2 in individuals with normal body surface area as abnormal.^7^ However, as no published data identify PVR in individuals with extremes of body sizes, the authors opted for PVR >2.3 Wood unit as abnormal.^7^ Although consensus for an abnormal PVR was reached, the acceptable range of cardiac output/index was not defined. Recent publications have identified hemodynamic parameters associated with outcomes;^7^^,^^9^, ^10^, ^11^, ^12^ however, the supporting evidence defining the optimal Fontan pressure in relation to morbidity and mortality risk is still lacking, and, critically, which specific hemodynamic phenotypes may benefit from therapeutic interventions have yet to be identified.

Challenges also exist for accurately measuring cardiac output in FC. Neither Fick using assumed or calculated oxygen consumption nor thermodilution methods appear reliable.^6^ Further complicating matters, Fontan patients have multiple sources of blood supply to the lungs, including Fontan fenestrations, veno-venous or aortopulmonary collaterals, and pulmonary arteriovenous malformations. Similar problems plague the use of MR imaging in quantifying blood flow, as well as interference from prosthetic materials and claustrophobia. Patients with such issues are not currently suitable for MR-based assessment. Such findings suggest a need to explore novel methods, including body impedance technology.^13^^,^^14^ A peripherally inserted venous cannula can provide central venous pressure measurement,^15^ which can be combined with MR-based flow assessment for in-depth hemodynamic assessment.^16^

Although PVR has been linked with adverse outcomes in FC,^7^, ^8^, ^9^, ^10^, ^11^ optimal or “normal” values have yet to be defined. In Fontan patients, only 1 vascular territory stretches from the peripheral veins to the pulmonary capillary bed. The resistance or pressure gradient between the pulmonary artery and the inferior vena cava should ideally be zero. A pressure gradient across the Fontan conduit as little as 1 mm Hg can be relevant, and exercising the patient in the cath lab can significantly increase such a gradient to unmask a relevant stenosis.^17^ Ideally, the transpulmonary gradient should be measured as a difference between the mean pulmonary arterial pressure and the mean pulmonary capillary wedge pressure, as it provides true physiologically important hemodynamic information.^18^

Limited publications have shown prognostic benefit of assessing hemodynamics during exercise in FC.^11^^,^^17^^,^^19^ Exercise testing can help to identify high-risk patients, such as those with exaggerated rise in pulmonary artery pressure in response to an increase in cardiac output (>3 mm Hg/L/min), or patients with a blunted increase in cardiac output and a disproportionate rise in Fontan pressure.^19^ Such testing has mainly been limited to a handful of institutions and has generally been used for research purposes rather than for routine clinical care. The results of our survey study reflect the significant uncertainty in the use of exercise testing, and the resultant heterogeneity of practice, even among experts. Findings from this pilot project will hopefully stimulate further study into FC hemodynamics and how they impact clinical management.

### Limitations

Limitations of our survey include the use of a convenience sample and reliance on voluntary responses. Although the sample size was relatively small, the survey achieved a robust response rate (>50%) and included participants from multiple countries and continents. Most respondents practiced in academic settings, which aligns with the typical care environment for patients with complex CHD, such as FC, thereby reflecting current practice accurately. The study is unfortunately underpowered to study individual differences in management between geographic locales. Also, despite several precautions, we had no mechanism to prevent individuals from responding more than once.

## Conclusions

There is considerable variability in Fontan care practices due to a lack of strong evidence-based guidelines. Defining “normal” hemodynamics, optimal surveillance, and clear criteria for Fontan failure and intervention points is essential for improving outcomes. An international registry covering both pediatric and adult Fontan patients is crucial to establish lifespan norms, especially as the adult Fontan population grows and life expectancy improves.
