# Supplementary material for: Physician Perception of Fontan Failure, “Acceptable” Hemodynamics, Assessment, and Indications for Intervention—Results of a Multinational Survey
Source: CJC Pediatr Congenit Heart Dis. 2025 Mar 14;4(4):198–202. doi: 10.1016/j.cjcpc.2025.02.004 (PMC12859612; doi:10.1016/j.cjcpc.2025.02.004)

Supplemental Figure S1

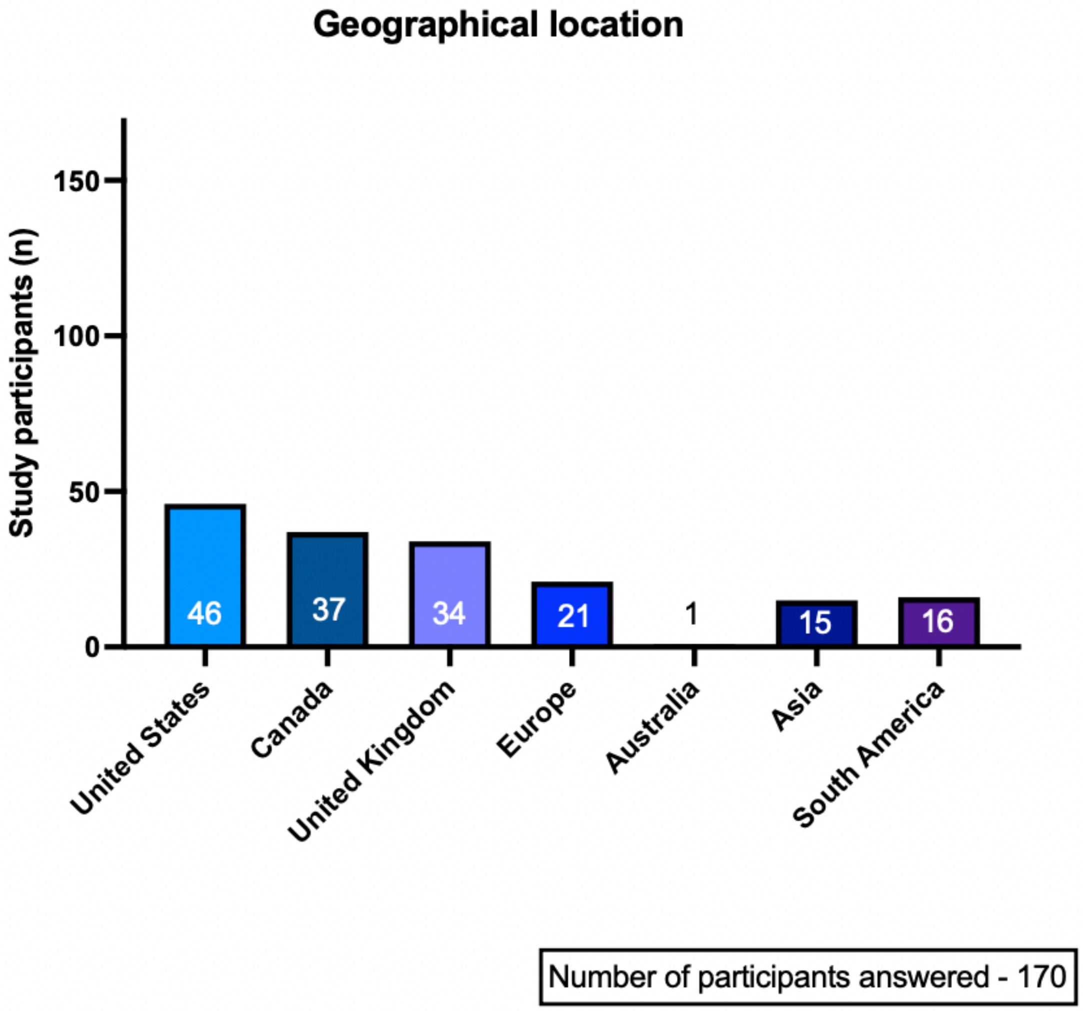

Supplemental Figure S2

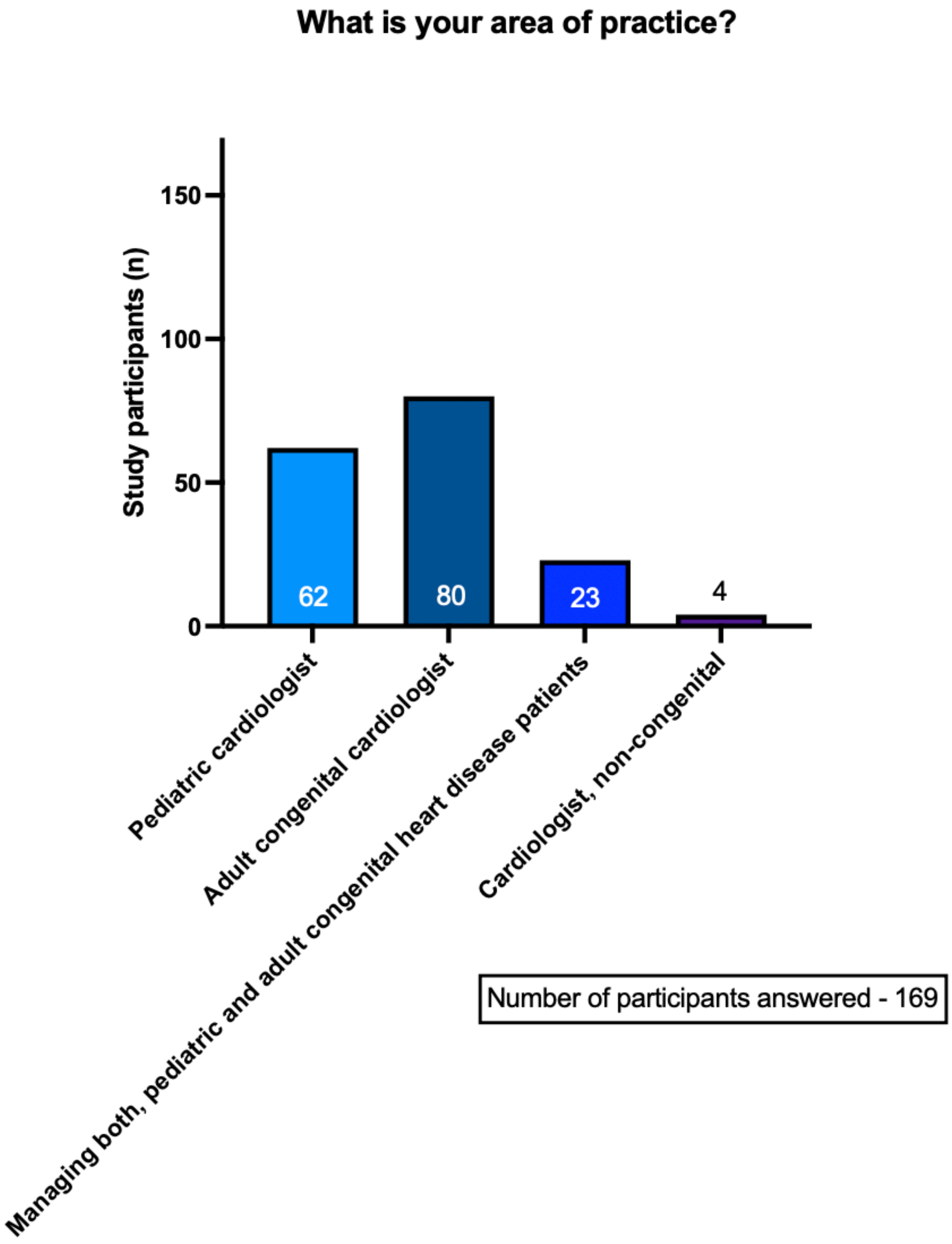

Supplemental Figure S3

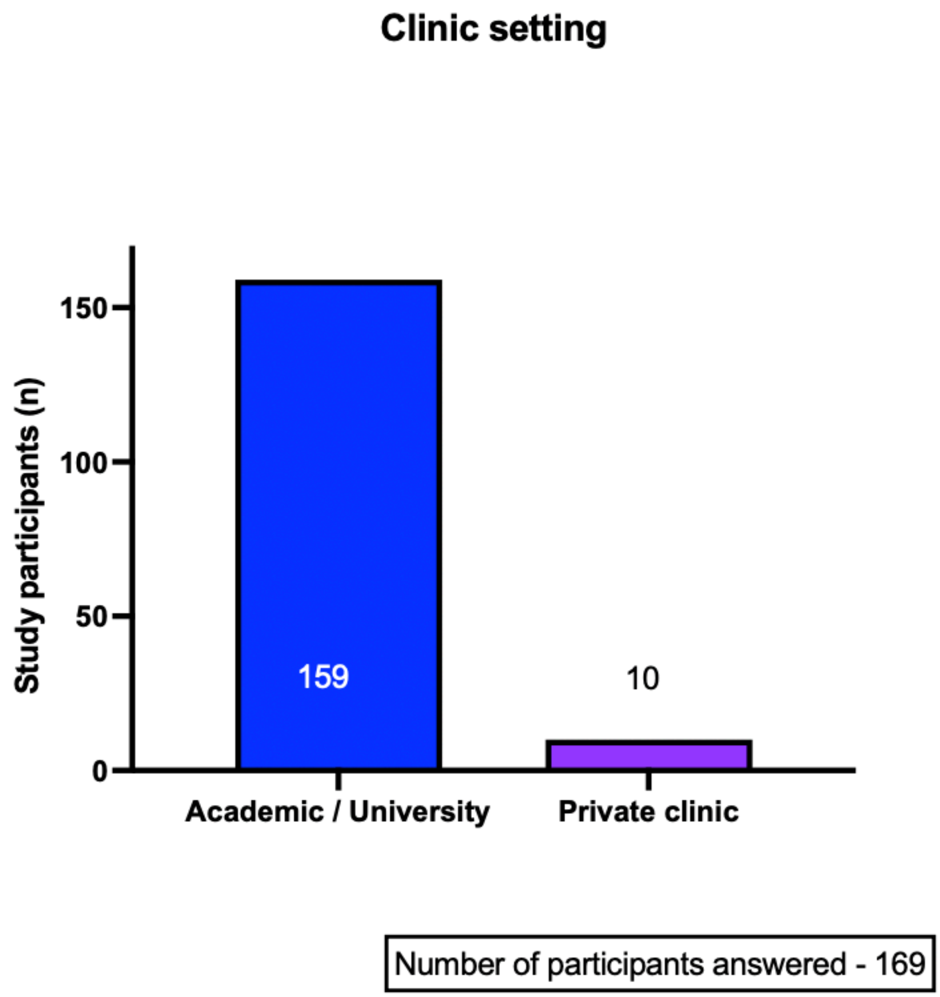

Supplemental Figure S4

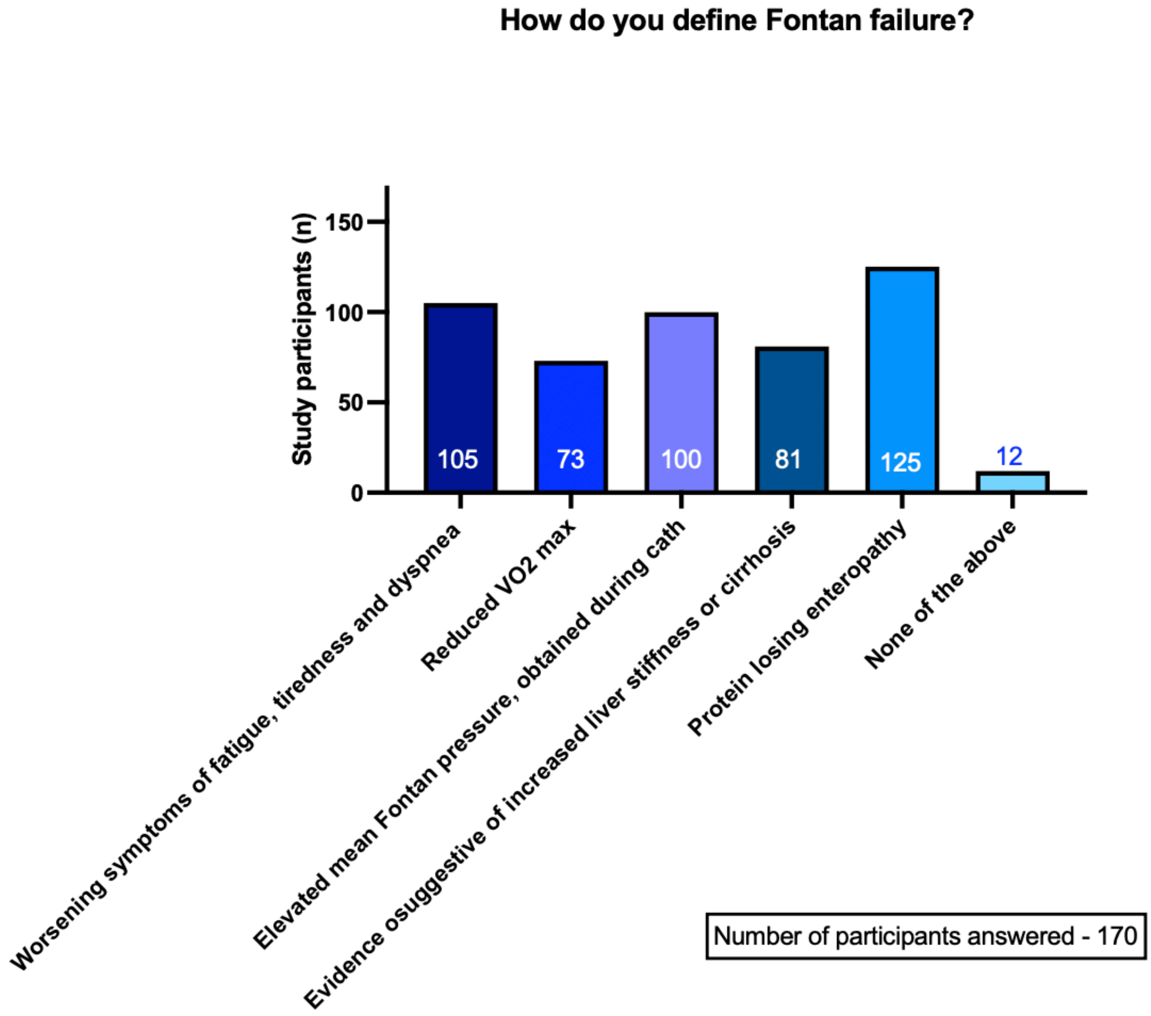

Supplemental Figure S5

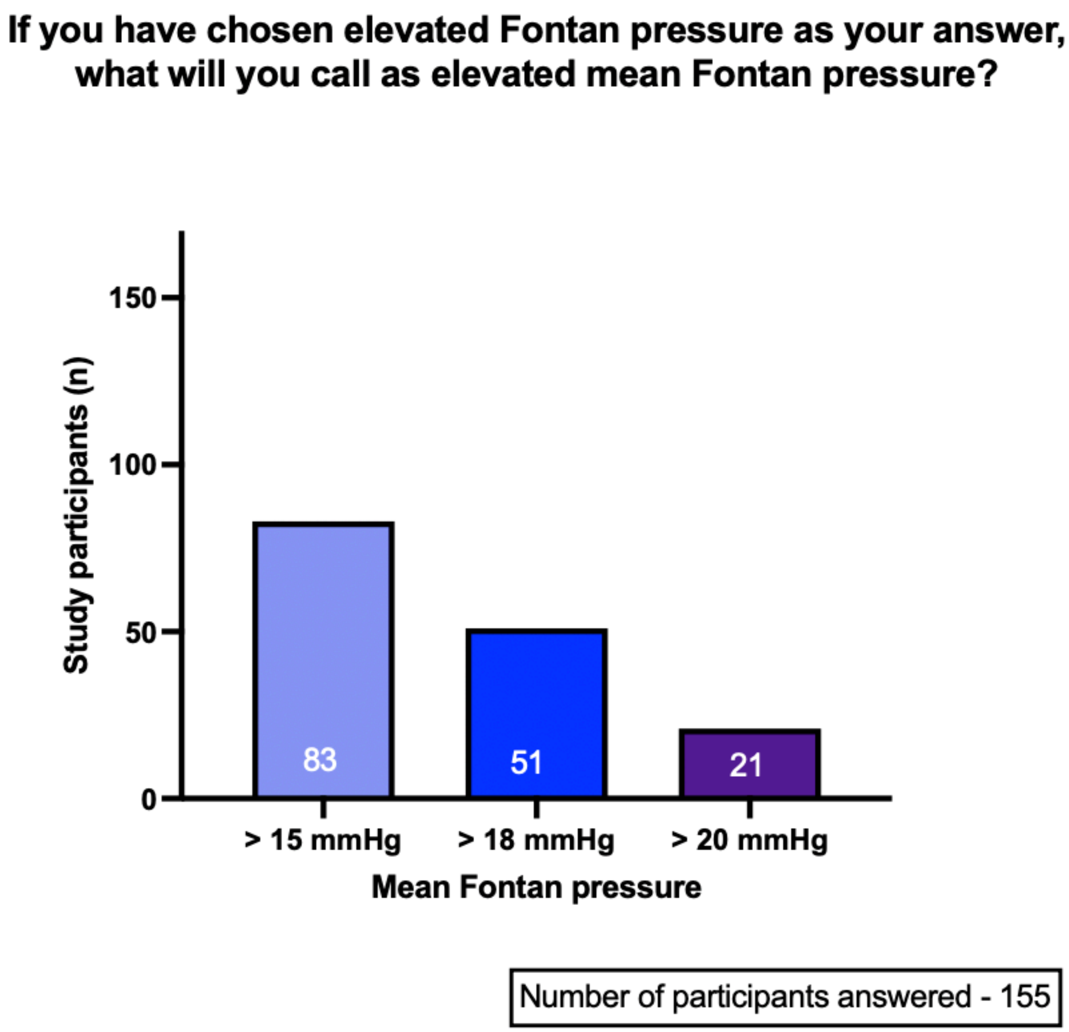

Supplemental Figure S6

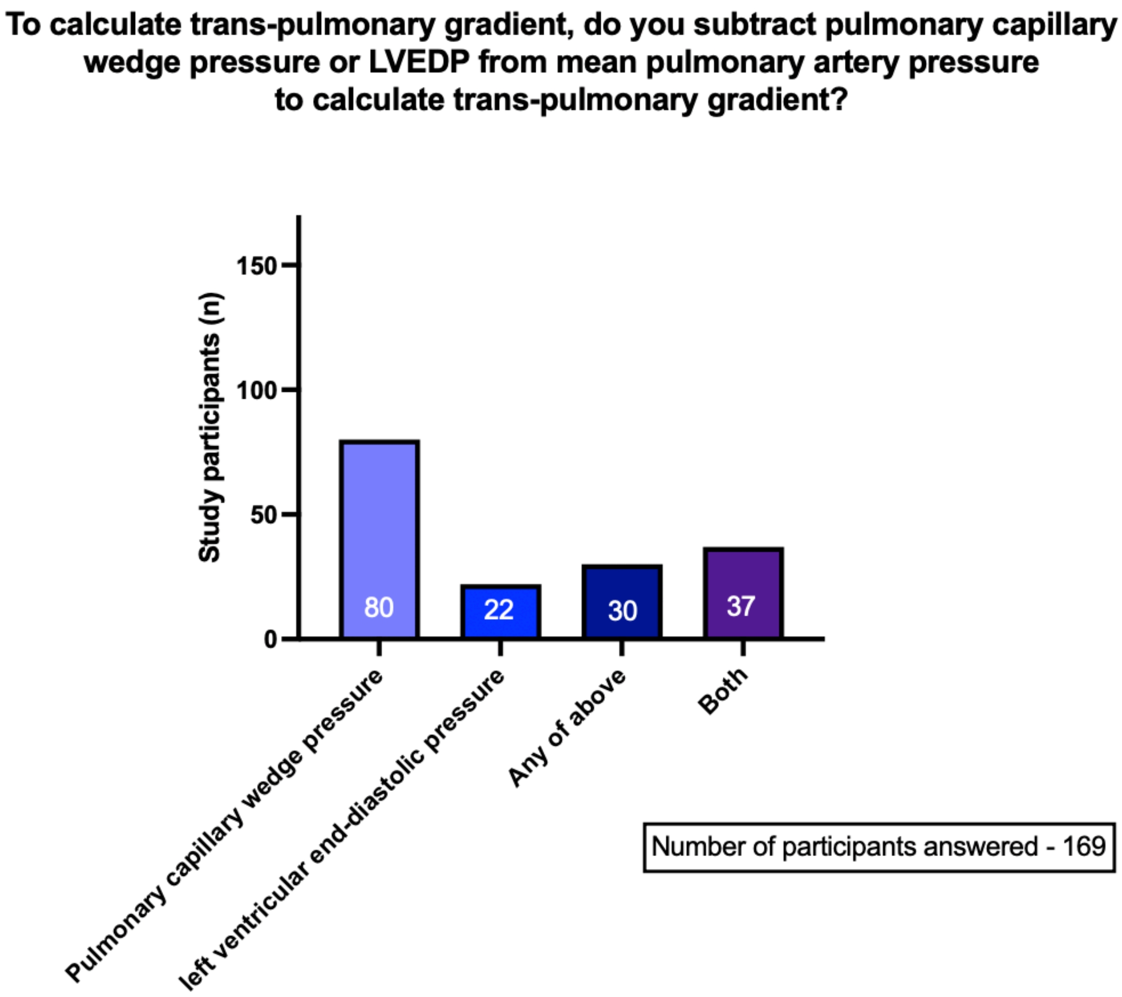

Supplemental Figure S7

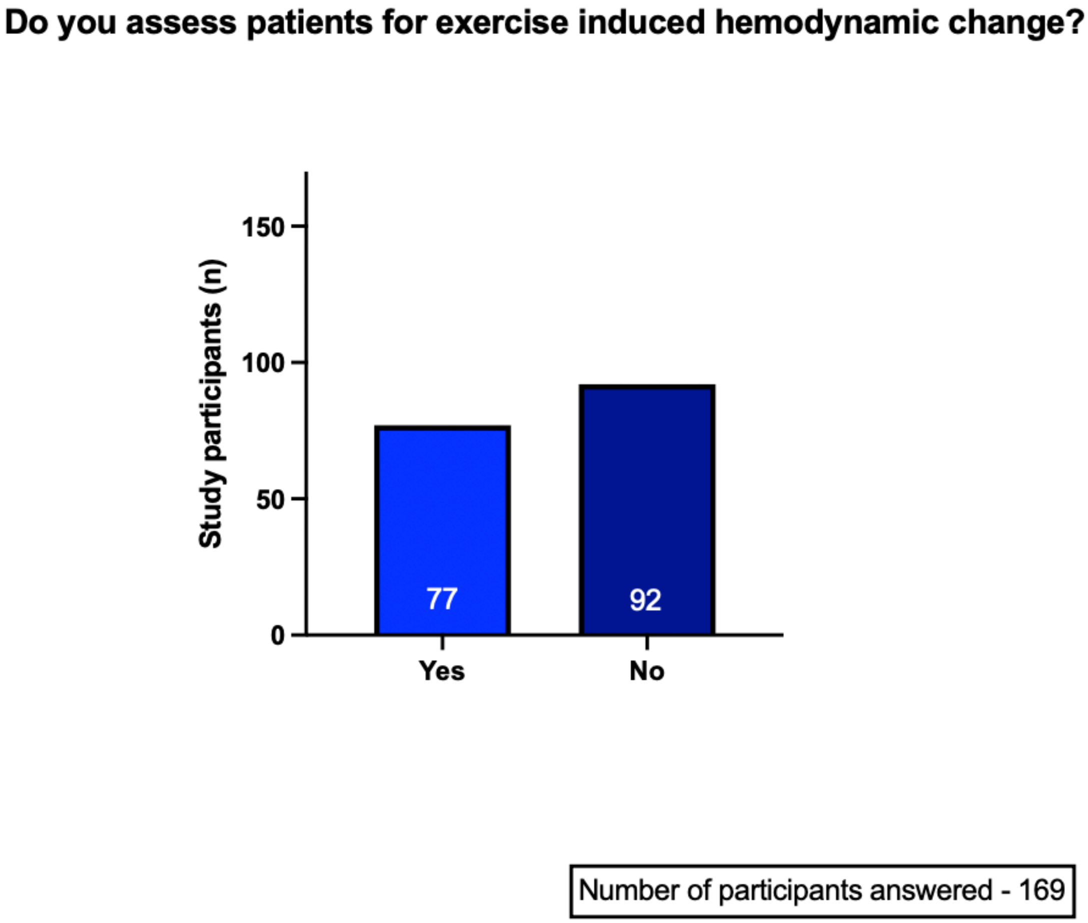

Supplemental Figure S8

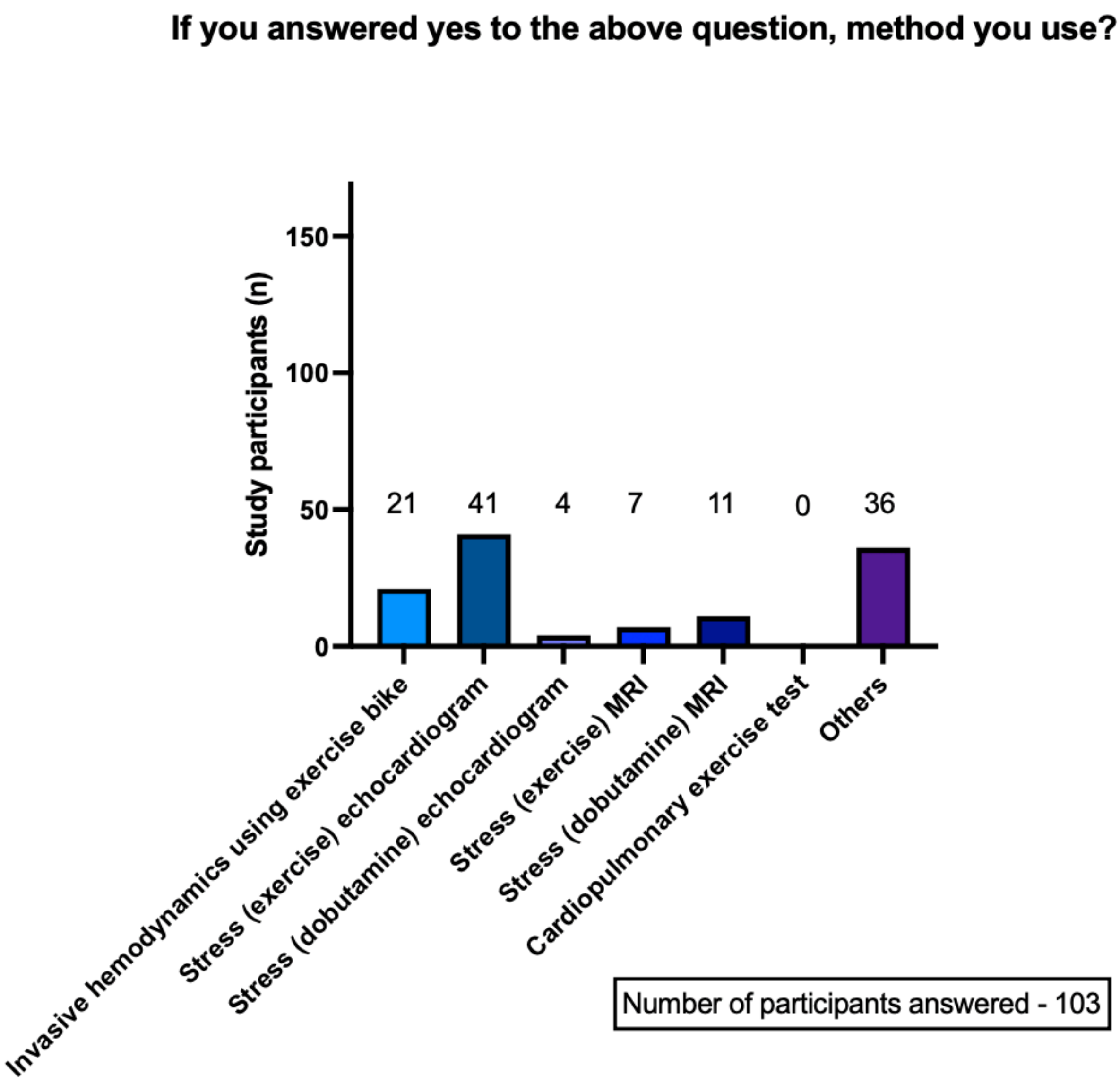

Supplemental Figure S9

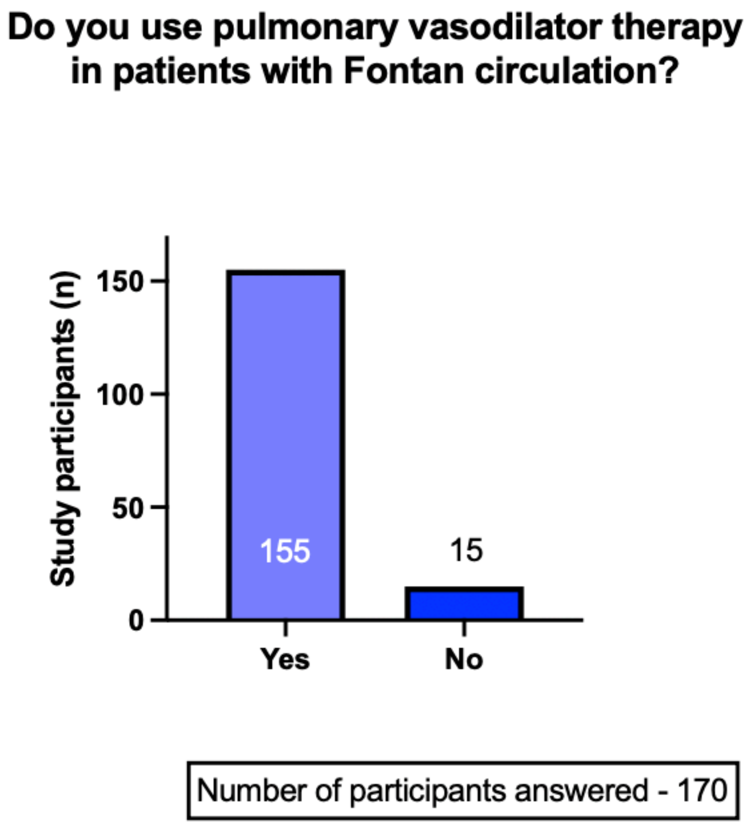

Supplemental Figure S10

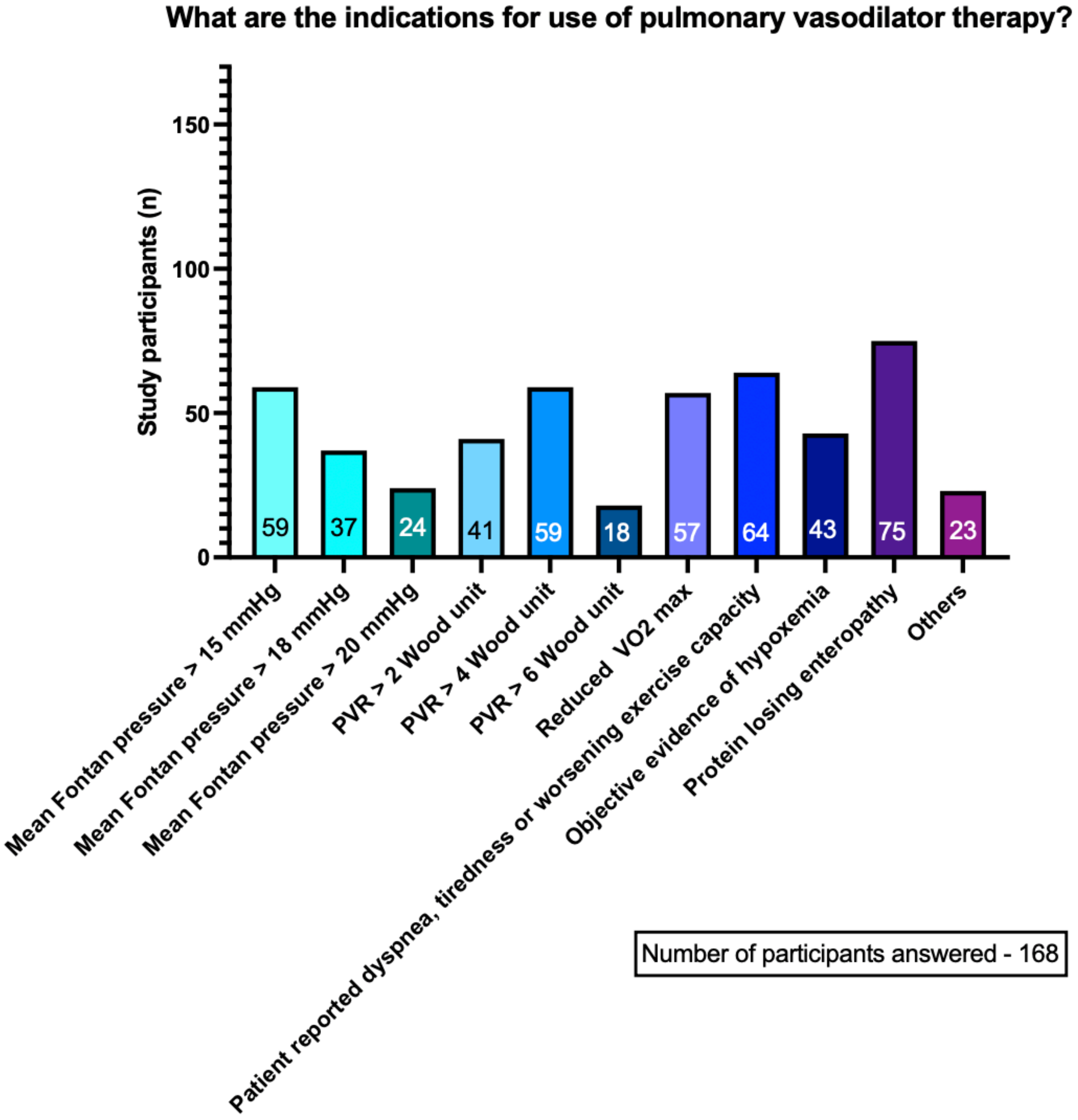

Supplement: Supplementary Figures 1-10 [file mmc1.pdf]
